# The Speed of Sound and Attenuation of an IEC Agar-Based Tissue-Mimicking Material for High Frequency Ultrasound Applications

**DOI:** 10.1016/j.ultrasmedbio.2012.02.030

**Published:** 2012-07

**Authors:** Chao Sun, Stephen D. Pye, Jacinta E. Browne, Anna Janeczko, Bill Ellis, Mairead B. Butler, Vassilis Sboros, Adrian J.W. Thomson, Mark P. Brewin, Charles H. Earnshaw, Carmel M. Moran

**Affiliations:** ∗ Centre for Cardiovascular Science, The University of Edinburgh, Edinburgh, United Kingdom; † Medical Physics, NHS Lothian, Royal Infirmary of Edinburgh, Edinburgh, United Kingdom; ‡ School of Physics, Dublin Institute of Technology, Dublin, Ireland; § Department of Clinical Physics, Royal London Hospital, Barts and the London NHS Trust, London, United Kingdom; || University of Cambridge, Cambridge, United Kingdom

**Keywords:** Ultrasound, High frequency, Tissue mimicking material, Speed of sound, Attenuation, Frequency dependence

## Abstract

This study characterized the acoustic properties of an International Electromechanical Commission (IEC) agar-based tissue mimicking material (TMM) at ultrasound frequencies in the range 10–47 MHz. A broadband reflection substitution technique was employed using two independent systems at 21°C ± 1°C. Using a commercially available preclinical ultrasound scanner and a scanning acoustic macroscope, the measured speeds of sound were 1547.4 ± 1.4 m∙s^−1^ and 1548.0 ± 6.1 m∙s^−1^, respectively, and were approximately constant over the frequency range. The measured attenuation (dB∙cm^−1^) was found to vary with frequency *f* (MHz) as 0.40*f* + 0.0076*f^2^*. Using this polynomial equation and extrapolating to lower frequencies give values comparable to those published at lower frequencies and can estimate the attenuation of this TMM in the frequency range up to 47 MHz. This characterisation enhances understanding in the use of this TMM as a tissue equivalent material for high frequency ultrasound applications.

## Introduction

The range of applications of high frequency ultrasound (>20 MHz) in life sciences has been increasing in recent years, specifically in the emerging fields of preclinical imaging (Maruvada et al. 2000; Goertz et al. 2005), intravascular ultrasound imaging (IVUS) (Rhee 2007), superficial tissue imaging (Vogt et al. 2007), ophthalmology (Silverman et al. 2008) and ultrasound biomicroscopy (Foster et al. 2000).

The purpose of a tissue mimicking material (TMM) is to closely mimic the speed of sound, attenuation and backscatter properties of soft tissue to evaluate new signal or image-processing algorithms (Kofler and Madsen 2001; MacGillivray et al. 2010) and to provide a reproducible way of assessing the image quality of diagnostic ultrasound (Browne et al. 2004; Shaw and Hekkenberg 2007). Various base materials have been used to produce either commercial or in-house TMM including agar, gelatine, n-propanol and oil (Duck 1990), urethane rubber, Zerdine^®^ and condensed milk (Browne et al. 2003).

To our knowledge, there is no commercially available TMM for the quality assessment of ultrasound scanners at high frequency range (Moran et al. 2011). One gelatine-based tissue mimicking material with spherical glass scatterers has been characterized from 2 to 60 MHz (Bridal et al. 1996), the attenuation of which depended nonlinearly with the frequency. The propagation speed and attenuation coefficient of a phantom tissue-like polymethylpentene was reported in the frequency range 20–70 MHz (Madsen et al. 2011). The nonlinear dependence of attenuation with frequency challenges the characterization of TMM at high frequencies. However, at the same time, there is increasing demand for the development of a tissue mimicking phantom, which is characteristic of both human and animal model tissue properties at high frequencies.

The IEC agar-based TMM characterised in this article was developed as part of an International Electromechanical Commission project (IEC 2001; Zeqiri and Hodnett 2010). The base ingredients of this TMM are water and glycerol. Agar is added to increase stiffness and its concentration, together with the glycerol, determines the speed of sound. Powders incorporating Al_2_O_3_ and SiC in different sizes are added to adjust the attenuation and scattering properties (Teirlinck 1997). It has been widely used, for example in flow phantom design (Ramnarine et al. 2001), in anthropomorphic phantoms (Inglis et al. 2006) and in breast phantoms (Cannon et al. 2011). The acoustic properties of this agar-based TMM and their temperature and frequency dependence have previously been investigated over the frequency range 2.25–15 MHz at an ambient temperature range of 10–35°C (Browne et al. 2003) and at an ultrasound frequency range 17–23 MHz over a temperature range of 22–37°C (Brewin et al. 2008). These authors showed that the speed of sound remained relatively constant with increasing frequency but that it increased with an increase in temperature while attenuation was shown to decrease with temperature. This TMM also showed acoustical stability over 2 years (Brewin et al. 2008) and exhibited a linear response of acoustic attenuation in the low frequency range 2–7 MHz (Browne et al. 2003), 6–15 MHz (Inglis et al. 2006) and 17–23 MHz (Brewin et al. 2008).

The aim of this article is to characterise the speed of sound and attenuation of the IEC agar-based TMM over the frequency range 10–47 MHz. Measurements were made using two independent systems, both employing a broadband reflection substitution technique (AIUM 1995).

## Materials and Methods

Measurement of the speed of sound and attenuation of the TMM were performed based on a broadband reflection substitution technique (AIUM 1995) using a Vevo 770^®^ preclinical ultrasound scanner (VisualSonics Inc., Toronto, Canada) in the University of Edinburgh and a scanning acoustic macroscope (SAM) system developed in-house in Dublin Institute of Technology (Cannon et al. 2011). Using the broadband reflection substitution technique, short duration, wideband transmitting pulses were used to acquire the data on the position and magnitude of the received sound pulse with and without the sample between a pulse-echo transducer and a specular reflector (AIUM 1995). As described below, the thickness of each sample at each acquisition was also calculated for measurements made using the Vevo 770 scanner.

### Manufacture of TMM test cells

**Fig. 1:**
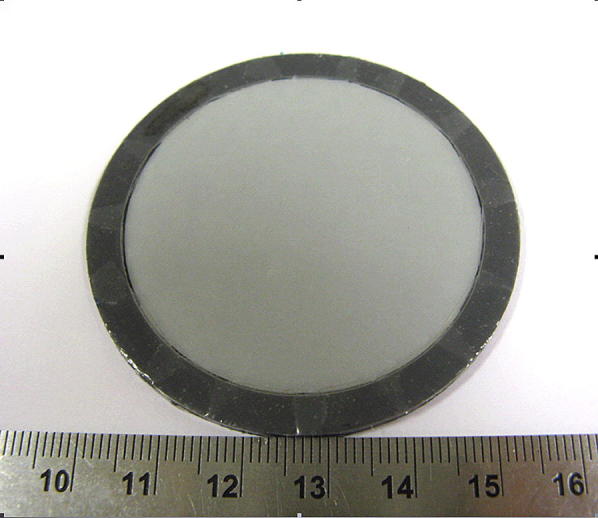
Tissue mimicking material (TMM) test cells.

The agar-based TMM used in this study was prepared following the method of Ramnarine et al. (2001), Browne et al. (2003) and Brewin et al. (2008). Due to the short focal length of the high frequency transducers (Table 1) that were employed on both measuring systems thin slices of TMM were manufactured. These were made by pouring a small volume of prepared TMM liquid at 46°C into PVC cylinder rings (inner diameter 4.8 cm, 2 mm height). Prior to pouring the TMM into the rings, a stretched layer of 14 – 16 μm thick Saran wrap (SC Johnson Inc., Racine, WI, USA) was glued to one rim of the ring. The TMM-filled ring was then left on a flat surface to set. Once the TMM was cooled to room temperature, before the upper layer of Saran wrap was stuck to the upper rim of the ring, approximately 0.2 mL of glycerol was placed on the TMM and spread over the surface to moisten it and ensure good acoustic coupling between the TMM and Saran wrap. Enclosure of the TMM samples in Saran wrap ensured that the glycerol did not leach from the TMM into the surrounding water-bath during measurements. Twelve TMM slices, which varied in thickness between 2 and 4 mm, were produced after cooling overnight. These TMM samples will be referred to as TMM test cells (Fig. 1). To account for the effect of the Saran wrap, water test cells of the same dimensions were also produced, in which the TMM was replaced by distilled water. Two batches of TMM were manufactured following the same protocol to assess the reproducibility of the process.

### Experimental set-up of Vevo 770^®^ preclinical ultrasound scanner

A Vevo 770^®^ preclinical ultrasound scanner and four single element transducers driven at 10% output power were used for the acoustic measurements. The power settings on the Vevo 770 scanner were varied between 3% and 100% over 16 discrete steps and 10% was set for our measurement. The frequency spectra of each transducer were calculated from the signal received by the membrane hydrophone placed at the focal position. From measurements made using a membrane hydrophone (data not included), 10% output power was considered a reasonable compromise between the generation of negligible nonlinear effects and adequate signal magnitude.

**Fig. 2:**
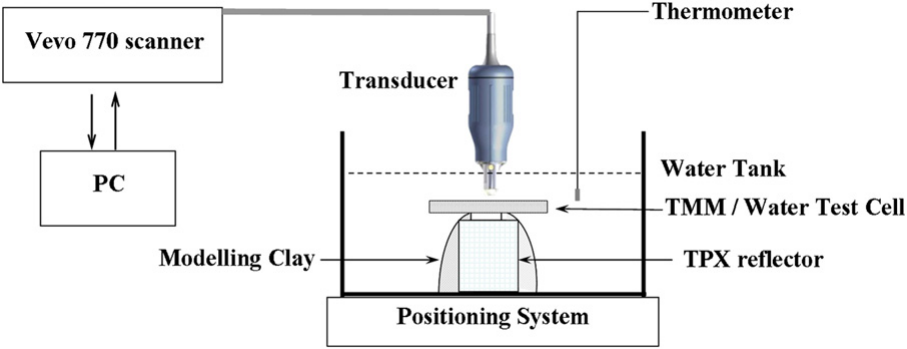
Schematic diagram of the experimental set-up of Vevo 770 scanner.

The nominal centre frequencies, focal lengths and the measured 3 dB band width of each of the transducers are given in Table 1. The 3 dB bandwidth of each transducer was calculated from the frequency spectra obtained from the reflected signal from a polished polymethylpentene (TPX) reflector (Boedeker Plastics, Shiner, TX, USA) placed at the focus of the transducer. Figure 2 shows a schematic diagram of the experimental set-up. The TMM test cell was placed and scanned in a water bath of air-free and distilled water and a TPX reflector was mounted beneath the test cell as a reference reflector. Modelling clay (Plasticine, Flair, UK) was used to secure the position of the reflector and to offset the position of the TMM test cell from the reference reflector. A three-dimensional positioning system (VisualSonics Inc., Toronto, Canada) with a step size of 0.1 mm was used to adjust the position of the transducer and the test cell. The tank containing the TMM test cell was seated on a bench-mounted adjustable two-dimensional (X, Y) rail system. The transducer was mounted on a Z positioning system. A physiological monitoring unit (VisualSonics Inc.) was used to measure the temperature of the water in real time. All the measurements were performed at 21°C ± 1°C.

The Vevo 770 scanner was operated in radio-frequency (RF) mode. The RF data in the region-of-interest (ROI) was sampled at a fixed frequency of 420 MHz. It was not possible to capture the RF data from the complete image frames so regions corresponding to the site of the relevant echoes were pre-selected. The RF data was downloaded from the scanner and calculations were performed off-line using MATLAB software (MATLAB 2009a, The MathWorks Inc., Natick, MA, USA).

### Measurement of speed of sound and thickness of TMM

**Fig. 3:**
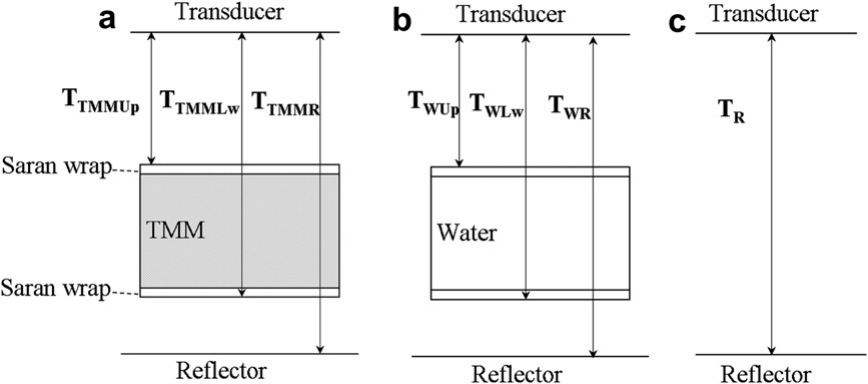
Diagram of the experiment and time intervals involved in calculations (not to scale). (a) Tissue mimicking material (TMM) test cell. (b) Water test cell. (c) Water path only. Definition of symbols can be found in Table 2.

The thickness of TMM and the speed of sound were calculated from the return time intervals of the pulse echoes from the front and rear surfaces of the TMM test cell and from the surface of the TPX reflector. Equations (1), (2) and (3) were used to derive the speed of sound [eqn (4)] and the thickness [eqn (5)] of the TMM. Figure 3 illustrates these time intervals schematically. The symbols in eqns (1)–(5) are defined in Table 2. The recorded temperature enabled the identification of the corresponding speed of sound in the water (Bilaniuk and Wong 1993).

In the experiment, the distance D_TR_ between the transducer and TPX reflector was fixed (Fig. 3a, b and c) and was equal to

(1) $${\text{D}}_{\text{TR}}=\left({\text{T}}_{\text{TMMUp}}+{\text{T}}_{\text{TMMR}}-{\text{T}}_{\text{TMMLw}}\right)\times \text{Vw}+\left[{\text{T}}_{\text{TMMLw}}-{\text{T}}_{\text{TMMUp}}-\left({\text{T}}_{\text{R}}-{\text{T}}_{\text{WR}}\right)\right]\times {\text{V}}_{\text{TMM}}+\left({\text{T}}_{\text{R}}-{\text{T}}_{\text{WR}}\right)\times \text{Vs}$$

(2) $${\text{D}}_{\text{TR}}=\left({\text{T}}_{\text{WUp}}+{\text{T}}_{\text{WR}}-{\text{T}}_{\text{WLw}}\right)\times \text{Vw}+\left[{\text{T}}_{\text{WLw}}-{\text{T}}_{\text{WUp}}-\left({\text{T}}_{\text{R}}-{\text{T}}_{\text{WR}}\right)\right]\times \text{Vw}+\left({\text{T}}_{\text{R}}-{\text{T}}_{\text{WR}}\right)\times \text{Vs}$$

(3) $${\text{D}}_{\text{TR}}={\text{T}}_{\text{R}}\times \text{Vw}$$

After re-arrangement of the equations, the speed of sound V_TMM_ and the thickness d_TMM_ of TMM in the TMM test cell are given by:

(4) $$V_{TMM}=\left(1+\frac{T_{WR}-T_{TMMR}}{T_{TMMLw}-T_{TMMUp}+T_{WR}-T_{R}}\right)V_{W}$$

(5) $$d_{TMM}=V_{TMM}\times \left[T_{TMMLw}-T_{TMMUp}-\left(T_{R}-T_{WR}\right)\right]$$

### Measurement of attenuation

The attenuation was calculated by subtraction of the frequency spectra of the RF signals from the reflector with the TMM test cell from that with the water test cell in the path. The attenuation of TMM relative to water α in the unit of $dB\cdot cm^{-1}$ was calculated using eqn (6):

(6) $$\alpha \left(x,y,f\right)=-\frac{20}{2d_{TMM}}\left[\log_{10}A\left(x,y,f\right)-\log_{10}A_{0}\left(x,y,f\right)\right]$$

where A(*x, y, f*) is the magnitude of the spectrum of the signal from the reflector with the TMM test cell in place (Fig. 3a), A_0_(*x, y, f*) is the magnitude of the spectrum of the signal from the reflector with the water test cell in place (Fig. 3b) and d_TMM_ is the thickness of TMM in the TMM test cell.

The attenuation of air-free distilled water is proportional to *f* ^2^ over the range 7.5–67.5 MHz (Pinkerton 1949). The attenuation in distilled water $\alpha_{w}$ at 20°C is 2.17 × 10^−3^ dB∙cm^−1^ ∙MHz^−2^ (Duck 1990).

The attenuation was calculated over the 3 dB bandwidth of each transducer. A polynomial curve fit was applied to the data using eqn (7).

(7) $$\alpha_{TMM}=\alpha +\alpha_{w}=af+bf^{2}$$

Where $\alpha_{TMM}$ is the absolute attenuation of TMM, *f* is the frequency and a and b are the coefficients of the polynomial function.

### Acquisition and analysis of acoustical data

Twelve TMM test cells were measured at three independent positions by each transducer. For each TMM test cell, the raw RF data of five lines (five positions) from 500 consecutive frames in three independent measurements was saved to the scanner and later transferred onto a PC and was analysed using MATLAB. The angular separation between adjacent RF acquisition lines was approximately 0.3° and so the lines were assumed to be parallel and perpendicular to the TPX reflector. The water test cell was scanned in a similar manner. For each position the mean and standard deviation of the thickness, speed of sound and attenuation were calculated.

For the reproducibility measurements, a second set of test cells made with a different batch of TMM were scanned in an identical manner.

### The speed of sound and attenuation using the SAM system

**Fig. 4:**
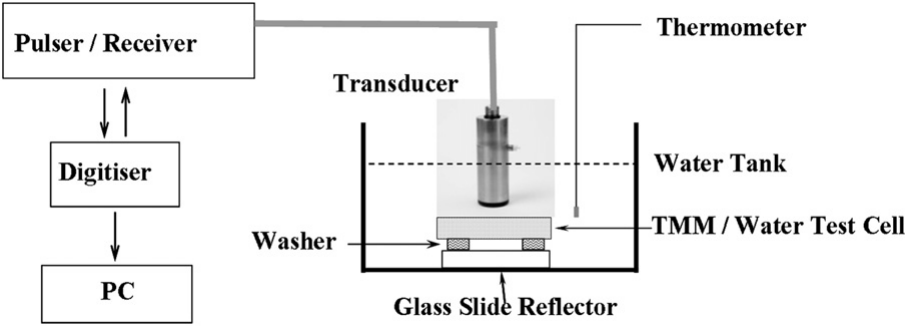
Schematic diagram of the scanning acoustic macroscope (SAM) system.

The SAM system (Fig. 4) used a broadband immersion transducer as both a transmitter and a receiver, which had frequency centred at 50 MHz with 1.27 cm focal length (V390-SU/RM; Olympus NDT Inc., Waltham, MA, USA). The measured 3 dB bandwidth of the transducer used in the SAM system was 10–33 MHz. For the SAM system, the same technique was employed for measuring the 3 dB band width as was used for the Vevo 770, but the reflector was a glass slide. A pulser-receiver (Model 5052PR; Panametrics, Waltham, MA, USA) with in-house software developed in LabView (National Instruments, Austin, TX, USA) (Cannon et al. 2011) manipulated the transmitting and receiving signals. The transmitted ultrasound pulses were perpendicular to the surface of a glass slide at the focal plane of the transducer. A computer with a data acquisition card (PCI–5144; National Instruments) then acquired and saved the reflected, digitised (250 MS s^−1^) signals (Model PCI-5114, Digitiser; National Instruments). Finally, the data was output to MATLAB for further calculations. A plastic washer of height 1.6 mm was attached on the glass slide to form a space between the test cell and reflector. All acoustic measurements were performed in degassed water at 20°C ± 1°C.

The experimental process of sample and reference measurements were similar to those employed using the Vevo 770 scanner. However, when using the SAM system, the complete RF signal from each line was collected. For the measurements of one TMM test cell, 10 independent positions were scanned and 10 consecutive pulses at each position were recorded for data analysis. The attenuation was calculated using eqn (6). The speed of sound of the TMM measured by the SAM system required a known thickness of TMM which was previously acquired from measurements using the Vevo 770 scanner. The speed of sound of the TMM, V_TMM_, was calculated using eqn (8) (AIUM 1995):

(8) $$V_{TMM}=\frac{V_{W}}{1+\Delta t\frac{V_{W}}{2d_{TMM}}}$$

where d_TMM_ is the known thickness of the TMM test cell measured by Vevo 770 scanner, V_w_ is the speed of sound in water and Δt is the measured time shift between T_TMMR_ and T_WR_.

## Results

The mean thickness values of each of the 12 TMM test cells measured by all the Vevo 770 transducers are listed in Table 3 and show a maximum variation of 0.06 mm.

Table 4 shows the measured TMM speed of sound and standard deviation. The mean values were found to be 1547.4 ± 1.4 m∙s^−1^ and 1548.0 ± 6.1 m∙s^−1^ measured by the Vevo 770 scanner and SAM system, respectively. Both results show good consistency and do not vary significantly over the frequency range 10–47 MHz. The acoustical properties from a second batch of six TMM test cells were also measured by the Vevo 770 scanner. The measured speed of sound was 1544.4 ± 1.0 m∙s^−1^.

**Fig. 5:**
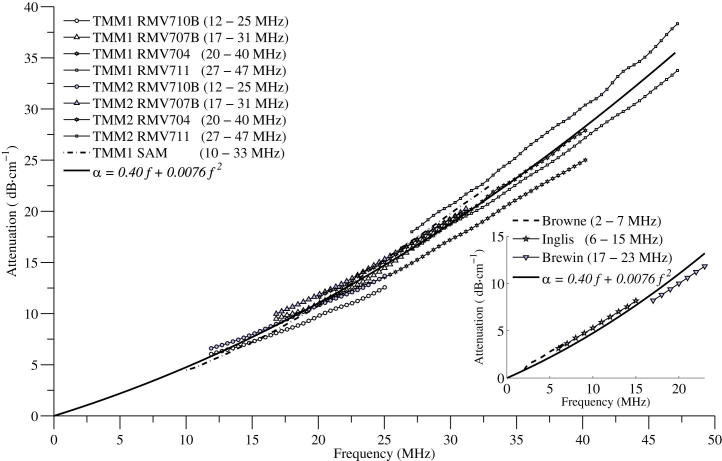
The polynomial curve-fit of the entire attenuation data set as a function of frequency (data set: both batches of tissue mimicking material (TMM) measured by the Vevo 770 scanner and SAM system in the frequency range of 10–47 MHz and the attenuation (compensated for attenuation of water) of TMM in 2–7 MHz (Browne et al. 2003), 6–15 MHz (Inglis et al. 2006) and 17–23 MHz (Brewin et al. 2008)). TMM1 is the first batch and TMM2 is the second batch. Inset displays the attenuation (corrected for the attenuation of water) at these lower frequencies.

**Fig. 6:**
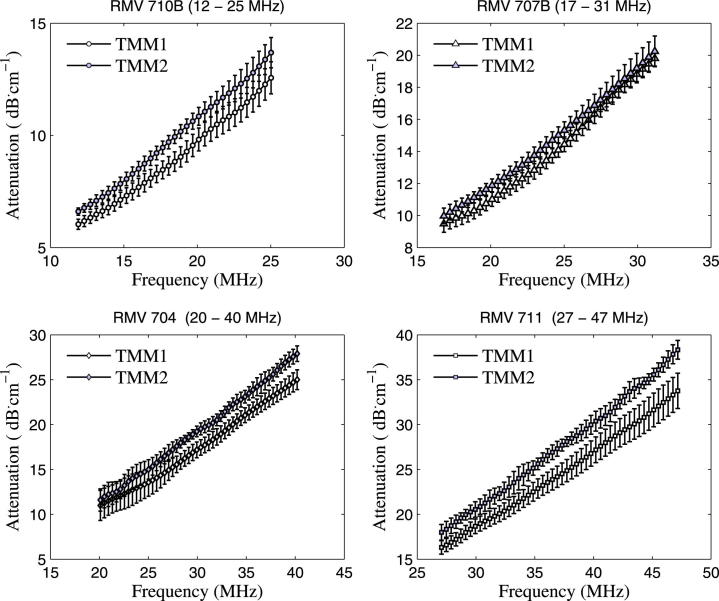
Mean and standard deviation of attenuation values of the two batches of tissue mimicking material (TMM) as a function of frequency measured by transducers 710B, 707B, 704 and 711.

Figure 5 shows the polynomial fitting curve with the measured attenuation of the two batches of TMM, demonstrating that the absolute attenuation in TMM increases with increasing frequency. This polynomial function 0.40*f* + 0.0076*f*^2^ was calculated to be the best-fit of all the relevant attenuation vs. frequency data available for this TMM from 2 to 47 MHz when the previous attenuation measurements (Browne et al. 2003; Inglis et al. 2006; Brewin et al. 2008) were compensated for the attenuation of water. The inset in Figure 5 represents the previous attenuation results (corrected for the attenuation of water) and the fitting curve at these lower frequencies. The mean attenuation and standard deviation obtained with each transducer and for both batches of TMM are presented in Figure 6. The polynomial function fitting all the attenuation curves from batch 1 and batch 2, measured by transducers 710B, 707B, 704, 711 and the SAM system are listed in Table 5 and their goodness of fit by R^2^ are shown to be greater than 0.99 for all fits.

## Discussion

This article reports for the first time acoustical measurements of the IEC agar-based TMM at ultrasound frequencies higher than those routinely used in clinical practice. We have shown that the measurements of speed of sound and attenuation at these frequencies are consistent with results at lower frequencies. We did not attempt to separate the attenuation into the separate components of absorption and scattering but measured the overall attenuation of the TMM.

In this study, the thickness of the TMM test cells was calculated using the RF data rather than by using mechanical callipers as the thin slices of TMM used in this study were easily compressed by the callipers. The small variation in the measured thickness at different sites on individual TMM slices is likely due to slight variations in the flatness of surface of TMM.

There are generally two approaches to test the thickness and speed of sound of a compressible and irregular shaped object using ultrasound. The first is to test the unsealed object in its preserving liquid, which requires a known speed of sound of the preserving liquid over the relevant ultrasound frequency range. The second is to test the sealed object in water, since the acoustic properties of water have been extensively investigated across a wide ultrasound frequency range. The method described in this article adopted the second technique. This method of measuring the speed of sound in the TMM is relative and based on the published data of absolute speed of sound values in water (Bilaniuk and Wong 1993) and described by AIUM (AIUM 1995). However, Lubbers and Graaff (1998) have suggested that the speed of sound measurements in water made by Del Grosso and Mader (1972) give more reproducible results. The discrepancy of speed of sound in pure water between the work of Del Grosso and Mader (1972) and Bilaniuk and Wong (1993) between 19–22°C is smaller than 0.02 m∙s^−1^ and, therefore, is smaller than the experimental error associated with this study.

The measured mean speed of sound in TMM in this study is comparable to the previously measured value of the speed of sound in TMM at lower frequencies. The thickness d_TMM_ used in the calculations of the SAM system was a mean thickness measured by the Vevo 770 scanner over 15 sites. Unlike the measurements made using the Vevo 770 when individual thickness measurements were used for each sampling position, a mean thickness for each TMM test cell (Table 3) was used in the measurement from the SAM system. This mean thickness value may contribute to the variation in speed of sound measurements by the SAM system.

In Figure 6 for all transducers, the attenuation of the second batch of TMM was shown to be higher than the first batch. The difference in mean attenuation between the two batches of TMM was found to be largest for the 711 transducer with a maximum difference of 5 dB∙ cm^−1^ at higher frequencies. The transducers 710B, 707B and 704 demonstrated differences in mean attenuation of less than 3 dB∙ cm^−1^.

The reflections from the TMM test cell interfaces contributed to the uncertainty on the attenuation measurements. Based on the transmission coefficients of TMM and water, a theoretical uncertainty due to the four water-TMM boundaries was calculated to be less than 0.008 dB∙cm^−1^∙MHz^−1^ over the frequency range of these experiments and so lay within experimental error of the attenuation measurements.

In addition, we have also quantified the uncertainties in the attenuation measurements due to the Saran wrap interfaces on the test cells. We performed two substitution technique experiments using similar methods to those described previously. In the first experiment, the frequency spectra from the TPX reflector with a TMM test cell in the ultrasound path was subtracted from the frequency spectra with the same TMM test cell uncovered *i.e*., without Saran wrap. In the second substitution experiment, the frequency spectra from the TPX reflector with and without a water test cell (water encased in Saran wrap) were subtracted. The difference between these two spectra was due to the difference in reflection coefficients at the Saran wrap interfaces in these experiments. The maximum difference in these spectra was found to be less than 0.11 dB∙cm^−1^∙MHz^−1^, which is comparable to the standard deviation of the absolute attenuation value of ultrasound through TMM as shown in Figure 6.

Polynomial functions were applied to the attenuation curves from each of the transducers, because the attenuation of water has been shown to be proportional to *f* ^2^ and the attenuation of TMM has been shown to vary linearly with frequency at low frequency range. Higher order terms, such as *f*^3^ were excluded as their coefficients proved to be very small (10^−5^) compared with that of the lower order terms. From Table 5, it can be seen that the coefficients of the linear terms varied between 0.4 dB∙cm^−1^∙MHz^−1^ and 0.5 dB∙cm^−1^∙MHz^−1^ for the four Vevo 770 transducers. The coefficients of quadratic term are not negligible. This indicates that with increasing frequency, TMM no longer attenuates linearly.

Furthermore, by combining all the data obtained from each of the transducers and the SAM system and including data at lower frequencies by other authors, a polynomial fit was applied to all the published data on this TMM from 2–47 MHz. The polynomial fit was found to be of the form 0.40 *f* + 0.0076 *f ^2^* and agreed well with the earlier measurements in the frequency range of 2–7 MHz (Browne et al. 2003) and 6–15 MHz (Inglis et al. 2006) and 17–23 MHz (Brewin et al. 2008). Moreover, at frequencies less than 15 MHz, this polynomial approximated a linear fit to the data in the form of 0.47*f* + 0.32.

## Conclusion

Published data on the acoustic properties of human and animal tissue at high frequency ultrasound (20–70 MHz) is limited and concentrated in the region of vascular tissues (Lockwood et al. 1991; Saijo et al. 1998), blood (Treeby et al. 2011), skin tissues (Moran et al. 1995; Huang et al. 2007) and bovine tissue (Maruvada et al. 2000). In this study the acoustical properites of an IEC agar-based TMM were measured from 21–47MHz to determine if such material has potential to be functioned as a suitable tissue mimic at high frequencies. The measured speeds of sound of an IEC agar-based TMM measured by the Vevo 770 scanner and SAM system were found to be 1547.4 ± 1.4 m∙s^−1^ and 1548.0 ± 6.1 m∙s^−1^. These values are consistent with the results in earlier studies by Browne et al. (2003) over the range 2.25–15 MHz and Brewin et al. (2008) over the range 17–23 MHz. The attenuation in agar-based TMM was shown to increase with increasing frequency and is comparable to previous results when it extrapolates to the low frequency range. However, at higher frequencies, the relationship between attenuation and frequency was shown to be nonlinear. A unifying polynomial function 0.40*f* + 0.0076*f^2^* was derived both based on the data generated in this study and on previously published data and was shown to be able to estimate the attenuation of this agar-based TMM in the frequency range 2–47 MHz. This characterisation of the TMM at frequencies greater than 20 MHz allows this IEC agar-based TMM to be potentially used in high frequency applications.

## Figures and Tables

**Table 1:** Characteristics of high frequency transducer

Transducer model RMV	710B	707B	704	711
Central frequency (MHz)	25	30	40	55
Focal length (mm)	15	12.7	6	6
Measured 3 dB band width (MHz)	12–25	17–31	20–40	27–47

The central frequency and focal length measurements are defined by manufacturer’s literature. The 3 dB bandwidth was measured from the frequency spectrum.

**Table 2:** Definitions of the symbols involved in the eqns (1)–(5)

T_TMMUp_	Time required for ultrasound pulse to travel from the transducer to the upper surface of the Saran wrap of the TMM test cell
T_TMMLw_	Time required for ultrasound pulse to travel from the transducer to the lower surface of the Saran wrap through the TMM test cell
T_TMMR_	Time interval between the transducer and surface of the TPX reflector through the TMM test cell
T_WUp_	Time required for ultrasound pulse to travel from the transducer to the upper surface of the Saran wrap of the water test cell
T_WLw_	Time required for ultrasound pulse to travel from the transducer to the lower surface of the Saran wrap through the water test cell
T_WR_	Time interval between the transducer and surface of the TPX reflector through the water test cell
T_R_	Time interval between the transducer and surface of the TPX reflector through the water only
D_TR_	Distance between the transducer and TPX reflector
Vw	Speed of sound in water
V_TMM_	Speed of sound in TMM
Vs	Speed of sound in Saran wrap
d_TMM_	Thickness of TMM in the TMM test cell

TMM = tissue mimicking material; TPX = polymethylpentene.

**Table 3:** The mean and standard deviation (in brackets) of thickness of 12 TMM test cells measured by Vevo770 scanner

Samples	1	2	3	4	5	6
Thickness (mm)	2.63 (0.02)	2.67 (0.04)	2.72 (0.03)	2.54 (0.02)	2.68 (0.02)	3.03 (0.03)

Samples	7	8	9	10	11	12
Thickness (mm)	3.17 (0.03)	2.55 (0.03)	2.96 (0.03)	2.47 (0.02)	2.75 (0.02)	2.72 (0.06)

TMM = tissue mimicking material.

**Table 4:** The mean and standard deviation (in brackets) of speed of sound (m∙s^−1^) of 12 TMM test cells measured by the four transducers of Vevo 770 scanner and SAM system

Transducer sample	710B	707B	704	711	SAM
1	1547.8 (0.5)	1547.6 (0.8)	1546.6 (4.1)	1546.9 (1.4)	1553.5 (2.7)
2	1548.3 (0.6)	1548.4 (0.9)	1547.9 (3.0)	1547.9 (2.0)	1552.1 (2.5)
3	1544.1 (1.5)	1545.7 (0.2)	1543.5 (0.4)	1547.2 (0.5)	1553.6 (2.4)
4	1547.4 (1.1)	1546.6 (1.1)	1548.3 (1.6)	1547.9 (1.0)	1554.8 (2.5)
5	1546.6 (1.6)	1547.0 (0.6)	1545.9 (2.0)	1546.6 (1.1)	1548.6 (5.6)
6	1546.1 (0.4)	1545.8 (0.5)	1546.7 (1.5)	1546.4 (0.3)	1549.5 (2.8)
7	1547.2 (0.4)	1547.0 (0.4)	1545.2 (1.5)	1547.4 (1.6)	1550.2 (2.5)
8	1547.8 (0.7)	1547.9 (0.6)	1547.3 (1.7)	1548.8 (0.5)	1539.6 (5.3)
9	1547.5 (0.5)	1547.8 (0.4)	1545.0 (1.3)	1548.3 (0.7)	1539.3 (3.3)
10	1547.4 (0.2)	1549.0 (1.0)	1547.6 (1.8)	1548.1 (1.4)	1542.6 (3.3)
11	1548.4 (0.5)	1550.2 (0.8)	1546.7 (2.7)	1548.1 (1.5)	1546.7 (3.0)
12	1548.4 (0.6)	1551.2 (0.8)	1549.4 (2.0)	1548.7 (2.3)	1545.9 (3.3)

TMM = tissue mimicking material; SAM = scanning acoustic macroscope.

**Table 5:** The polynomial fit $\left(\alpha \;=\;\text{a}f+\;\text{b}f^{2}\right)$ of the attenuation of the two batches of TMM measured by Vevo 770 scanner and the SAM system

Transducer	710B	707B	704	711	Combination	SAM
Batch 1	0.47*f* + 0.00090*f^2^*	0.40*f* + 0.0074*f^2^*	0.43*f* + 0.0048*f^2^*	0.45*f* + 0.0055*f^2^*	0.39*f* + 0.0069*f^2^*	0.32*f* + 0.011*f^2^*
Batch 2	0.53*f* + 0.00030*f^2^*	0.50*f* + 0.0048*f^2^*	0.46*f* + 0.0059*f^2^*	0.48*f* + 0.0069*f^2^*	0.39*f* + 0.0088*f^2^*	

TMM = tissue mimicking material; SAM = scanning acoustic macroscope.
